# State selective classical electron capture cross sections in Be^4+^  + H(1*s*) collisions with mimicking quantum effect

**DOI:** 10.1038/s41598-021-99759-y

**Published:** 2021-10-11

**Authors:** Iman Ziaeian, Károly Tőkési

**Affiliations:** 1 Institute for Nuclear Research (Atomki), Bem tér 18/c, 4026 Debrecen, Hungary; 2 Doctoral School of Physics, Faculty of Science and Technology, University of Debrecen, P.O. Box 400, 4002 Debrecen, Hungary

**Keywords:** Atomic and molecular collision processes, Theoretical nuclear physics

## Abstract

We present state-selective electron capture cross sections in collision between Be^4+^ and ground state hydrogen atom. The *n*- and *nl*-selective electron capture cross sections are calculated by a three-body classical trajectory Monte Carlo method (CTMC) and by a classical simulation schema mimicking quantum features of the collision system. The quantum behavior is taken into account with the correction term in the Hamiltonian as was proposed by Kirschbaum and Wilets (Phys Rev A 21:834, 1980). Calculations are carried out in the projectile energy range of 1–1000 keV/amu. We found that our model for Be^4+^ + H(1*s*) system remarkably improves the obtained state-selective electron capture cross sections, especially at lower projectile energies. Our results are very close and are in good agreement with the previously obtained quantum–mechanical results. Moreover, our model with simplicity can time efficiently carry out simulations where maybe the quantum mechanical ones become complicated, therefore, our model should be an alternative way to calculate accurate cross sections and maybe can replace the quantum–mechanical methods.

## Introduction

Beryllium is widely used as a first wall element of the fusion reactors^1^ because of its unique thermo-physical properties. So, due to wall erosion, Beryllium should be one of main impurity in fusion chamber^2^. The radiative decay of excited impurity ions can be the source for the energy loss of the plasma and can cool the plasma. These radiative decays can be analyzed by the electron capture recombination spectroscopy (CXRS). Therefore, the exact knowledge of electron capture cross sections in collisions between Be ions and hydrogen atoms is essential^3^. Due to the experimental difficulties, the experimental results for electron capture cross sections in Be^4+^  + H collisions are entirely lacking, but those were studied intensively theoretically in the past years. The total electron capture cross sections have been studied using various models and methods such as applying the quantum–mechanical molecular orbital close-coupling (QMOCC)^4^, the atomic orbital close-coupling (AOCC)^5^, the hyper spherical close-coupling (HSCC)^6^ models, using the solution of the time dependent Schrödinger equation (TDSE)^7^, the lattice time dependence Schrödinger equation (LTDSE)^8^, the classical over barrier model (COBM)^9^ and the classical trajectory Monte Carlo method^10,11^. The partial electron capture cross sections in the collision between Be and hydrogen atom have been also studied using different quantum–mechanical methods such as: QMOCC^4,12^, AOCC^5^, one-electron diatomic molecule (OEDM)^13^, and boundary corrected continuum intermediate state (BCCIS)^14^ models. It is worth noting that all the results have been published for projectile energy below 100 keV/amu. The calculation of the principle quantum number, *n*, dependent cross sections has been studied by Jorge et al.^15^ by solving the time-dependent Schrödinger equation with the GridTDSE package (GTDSE) numerically in the broad energy range between 1 keV/amu and 500 keV/amu.

In this work we present the electron capture cross sections into the bound states of the projectile in Be^4+^ + H(1*s*) collisions. We treat the collision dynamics classically using a three-body classical trajectory Monte Carlo (CTMC) and a three-body quasi classical Monte Carlo (QCTMC) model when the Heisenberg correction term is added to the standard CTMC model via model potential^16–21^. Since there is no experimental data available, our calculated cross sections are compared with the previous theoretical results.

## Results

For each collision energies, the calculation of the state selective electron capture cross sections requires to follow 10^7^ classical trajectories. At first, we tested three calculation schemes during our simulations since the Heisenberg correlation potential may influenced the obtained results significantly. These are the following: (1) target-centered, where the correction term is taken into account between the target electron the target nucleus, (2) projectile-centered, where the correction term is taken into account between the target electron the projectile. (3) Combined one, i.e., target and projectile centered when the correction term is taken into account between target electron and both the target nucleus and projectile.

**Figure 1:**
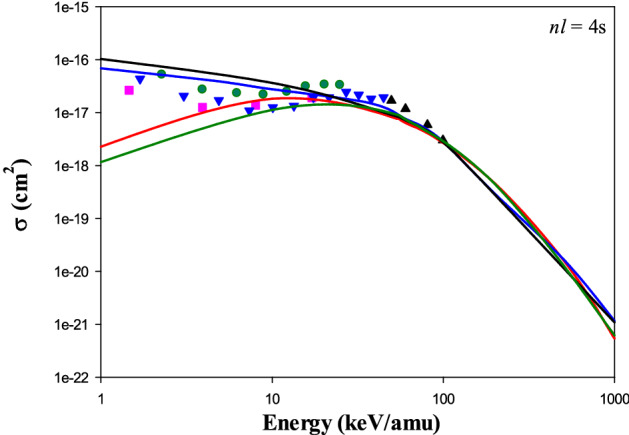
Electron capture cross sections into the 4*s* state of the projectile in Be^4+^  + H (1*s*) collision as a function of the impact energy. Solid red line: present CTMC results, solid black line: present *target-centered* QCTMC results, solid green line: present *projectile-centered* QCTMC results, solid blue line: present *target and projectile centered* QCTMC results, pink squares: AOCC results of Fritsch^5^, green circles: QMOCC results of Harel et al.^4^, black triangles: BCCIS results of Das et al.^14^, blue inverse triangles: OEDM results of Errea et al.^13^.

As an example Fig. 1 shows our CTMC and QCTMC results corresponding to the three calculation schema of the electron capture cross sections into the 4 s state of the projectile in Be^4+^  + H (1*s*) collision as a function of the impact energy. It can be seen that the effects of the correction term at lower energies are significant. While for the case of target-centered, the cross sections at lower incident energies are increasing compared to the standard CTMC results for the case of projectile-centered they are decreasing. The combination of the use of target- and projectile-centered corrections results increases the cross sections and we also obtained good agreement between our QCTMC results and previous full quantum mechanical results in the entire impact energy range. Therefore, in followings, for the calculation of the capture cross sections, we will use only the combination scheme.

Physically, due to the Heisenberg constraint, the electron cannot collapse to the target and projectile nucleus in the electron capture channel. To clarify this further, we calculated the electron capture probabilities as a function of the impact parameter.

**Figure 2:**
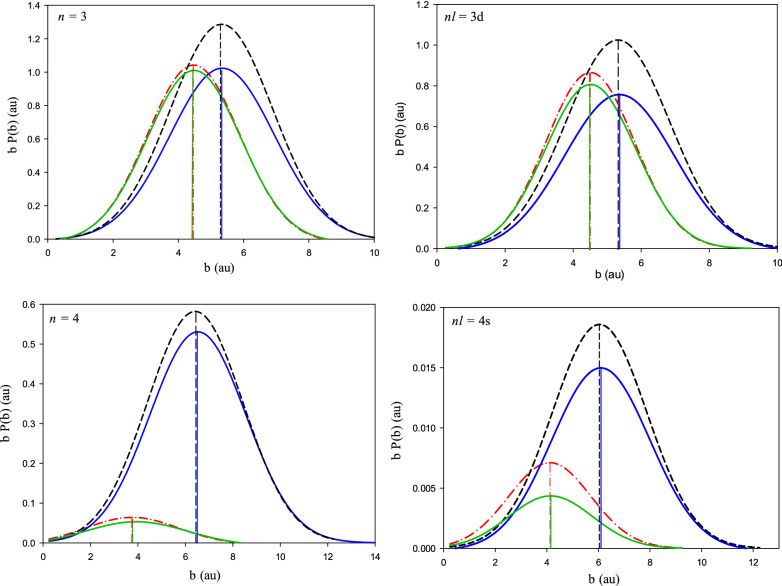
Probability for electron capture into *n* = 3, 4 and *nl* = 3d, 4*s* states of the projectile (multiplied by impact parameter) in Be^4+^  + H(1*s*), as a function of the impact parameter, at 10 keV/amu impact energy. Dash-dotted red line: present CTMC results, dash black line: present target-centered QCTMC results, solid green line: present projectile-centered QCTMC results, solid blue line: combination of target-projectile-centered.

Figure 2 shows the present CTMC and QCTMC results with the three calculation schemes of the electron capture probabilities into the specific *n* = 3, 4 and *nl* = 3d, 4*s* states of the projectile at 10 keV/amu impact energy in Be^4+^  + H(1*s*) as a function of impact parameter. The impact parameter dependent electron capture probabilities, *bP(b)*, were fitted by a Gaussian function. The peak maxima of the Gaussian fitting is also shown in Fig. 2. We note that the area under the curves is proportional to the state-selective electron capture cross sections. We found that the probability of electron capture is higher in target-centered QCTMC and lower in projectile-centered QCTMC model compared with the standard CTMC model. This behavior can be understood with the explanation of the acting forces between the interacting particles, $F = - dU/dr$. The attractive force between an electron and both of proton and positive projectile ion, is due to the Coulomb interaction and repulsive force is due to the Heisenberg correction term as follow:

1 $${F}_{Heisenberg}= -\left(\frac{{{\xi }_{H}}^{2}}{2{\alpha }_{H}{r}^{3}}+\frac{r{p}^{4}}{{{\xi }_{H}}^{2}}\right)exp\left\{{\alpha }_{H}\left[1-{\left(\frac{rp}{{\xi }_{H}}\right)}^{4}\right]\right\}$$

The attractive Coulomb force acts between the electron and positively charged, target and projectile, in the same way in all schemes. This force, most of the time of the collision, is much larger than ${F}_{Heisenberg}$. On the other hand, in the target-centered scheme, the repulsive force, ${F}_{Heisenberg}$, is toward the projectile, but on the contrary, this repulsive force is towards the target in projectile-centered mode. We note that this repulsive force, of course, does not show up in the standard CTMC model. According to the sum of the forces, the electron has the highest attraction to the projectile in the target-centered QCTMC and the least attraction to the projectile in the projectile-centered QCTMC. With this scenario, the case of CTMC is placed between the above two modes. Therefore, the probability of electron capture in projectile-centered QCTMC, CTMC, and target-centered QCTMC modes increases, respectively.

Another noteworthy point is that the peak maxima in CTMC and QCTMC projectile-centered cases are very close to each other and locate in lower impact parameters. This is also true in QCTMC target-centered and QCTMC combined target- and projectile-centered cases, except that the peak maxima are at higher impact parameters.

**Figure 3:**
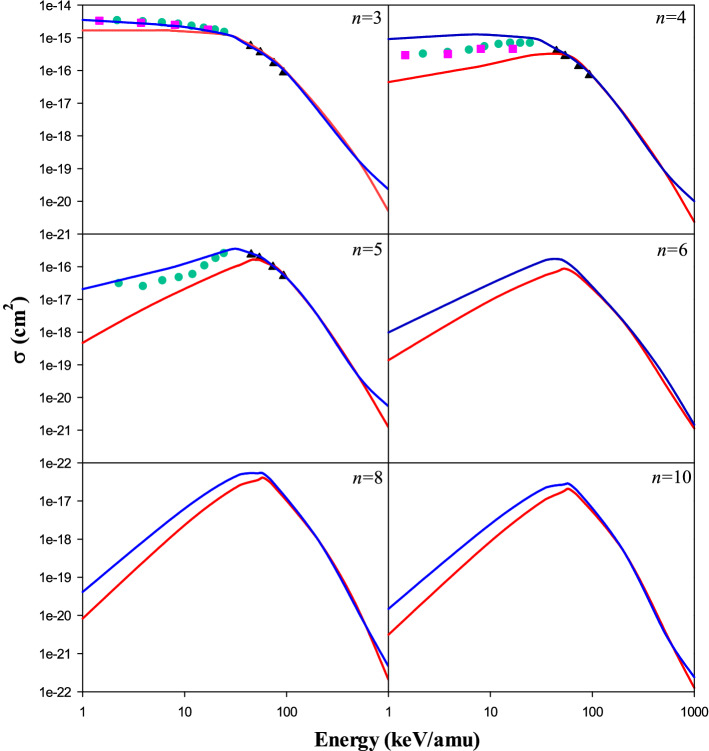
Electron capture cross sections into the *n* = 3, 4, 5 states of the projectile in Be^4+^  + H(1*s*) collision as a function of the impact energy. Solid red line: present CTMC results, solid blue line: present QCTMC results, pink squares: AOCC results of Fritsch^5^, green circles: QMOCC results of Harel et al.^4^, black triangles: BCCIS results of Das et al.^14^. Also, electron capture cross sections into *n* = 6, 8, 10 states are recommended.

Figure 3 shows the present CTMC and QCTMC results of the electron capture cross sections into the *n* = 3, 4, 5 states of the projectile in Be^4+^  + H(1*s*) collision as a function of the impact energy. The present classical results are compared with Fritsch^5^, Harel et al.^4^, and Das et al.^14^, as well. The QCTMC results are higher than the CTMC ones at low and intermediate impact energies. This difference is more significant in *n* = 4 and *n* = 5 states. The best matching between present CTMC and QCTMC is seen at high energies. In *n* = 3 and *n* = 5 states, the present QCTMC results agree well with the available quantum–mechanical approaches such as; QMOCC^4^, AOCC^5^, and BCCIS^14^.

The standard statistical error [see Eq. (10)] at 1000 keV/amu impact energy is around 4% in CTMC and QCTMC, respectively. Figure 3 also shows the cross sections for higher states where no previous data are available.

**Figure 4:**
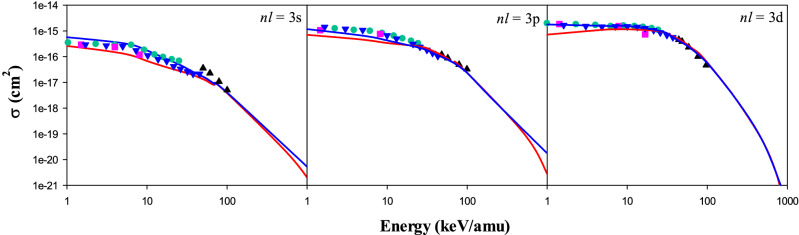
Electron capture cross sections into 3*s*, 3*p*, and 3*d* states of the projectile in Be^4+^  + H(1*s*) collision as a function of the impact energy. Solid red line: present CTMC results, solid blue line: present QCTMC results, pink squares: AOCC results of Fritsch^5^, green circles: QMOCC results of Harel et al.^4^, black triangles: BCCIS results of Das et al.^14^, blue inverse triangles: OEDM results of Errea et al.^13^.

Figure 4 shows our CTMC and QCTMC results of the electron capture cross sections into 3*s*, 3*p* and 3*d* states of the projectile in Be^4+^ + H(1*s*) as a function of the impact energy. The comparison is made with Fritsch^5^, Harel et al.^3^, Das et al.^14^, and Errea et al.^13^. The QCTMC model significantly improve the cross sections compared to the CTMC at low and intermediate impact energies. Moreover, the unique agreement is obtained between the present QCTMC results and (1) QMOCC results^4^ in the 3*s* state, (2) OEDM^13^, and BCCIS results^14^ in 3*p* state (3) QMOCC^4^, AOCC^5^, OEDM^13^, and BCCIS^14^ results in 3*d* state of the Be^3+^ at energies lower than 100 keV/amu, respectively.

**Figure 5:**
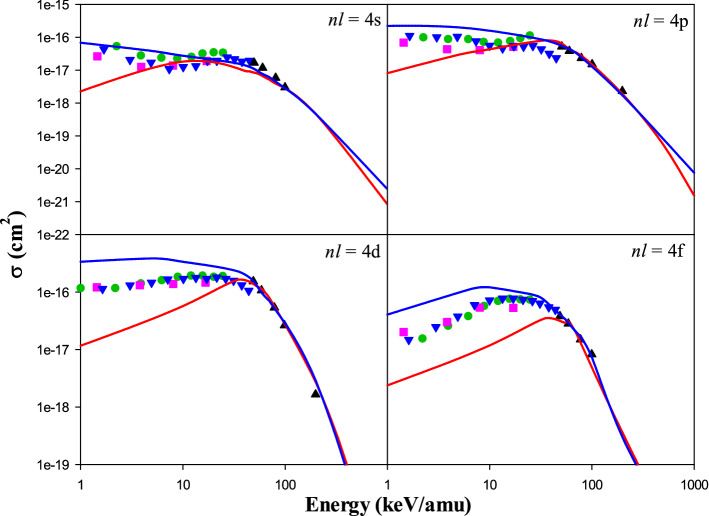
Electron capture cross sections into 4*s*, 4*p*, 4*d*, and 4*f* states of the projectile in Be^4+^  + H(1*s*) collision, as a function of the impact energy. Solid red line: present CTMC results, solid blue line: present QCTMC results, pink squares: AOCC results of Fritsch^5^, green circles: QMOCC results of Harel et al.^4^, black triangles: BCCIS results of Das et al.^14^, blue inverse triangles: OEDM results of Errea et al.^13^.

Figure 5 represents our CTMC and QCTMC results of the electron capture cross sections into 4*s*, 4*p*, 4*d*, and 4*f* states of the projectile in Be^4+^ + H(1*s*) as a function of the impact energy. We have compared the present classical results with the quantum–mechanical approaches such as QMOCC^4^, AOCC^5^, OEDM^13^, and BCCIS^14^. The QCTMC model remarkably increases the cross sections compared with the CTMC at low and intermediate energies. The difference between the present CTMC and QCTMC results at low energies gradually increases from 4*s* to 4*f* states. It can be seen that our CTMC results have the best agreement with the AOCC results of Fritsch^5^ in 4*s* and 4*p* states. Moreover, the QCTMC cross sections in 4*s* and 4*p* states have better agreement with the QMOCC results of Harel et al.^4^ and the OEDM results of Errea et al.^13^. However, both present classical results are in excellent agreement with the BCCIS results of Das et al.^14^ at intermediate energies. The standard statistical error for 4*s*, 4*p*, 4*d*, and 4*f* states is around 1.6% in the range of 1–500 keV/amu impact energies. At the same time, for the projectile energy range of 500–1000 keV/amu, the estimated uncertainties around 4%.

**Figure 6:**
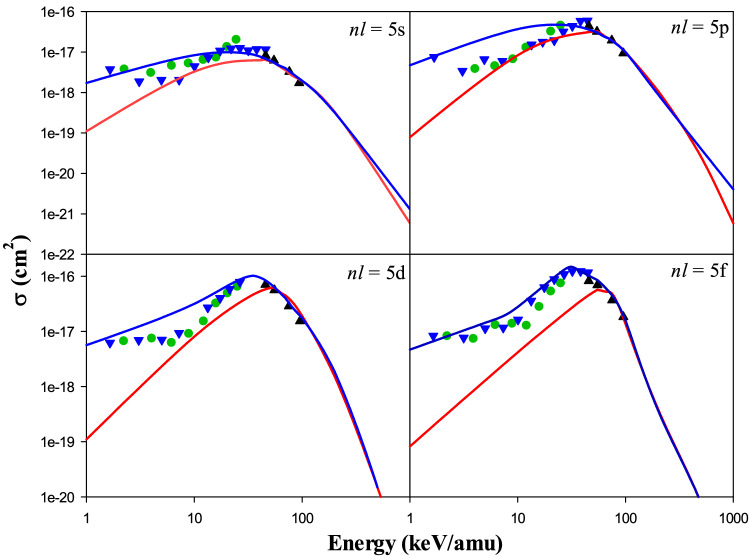
Electron capture cross sections into 5*s*, 5*p*, 5*d*, and 5*f* states of the projectile in Be^4+^  + H(1*s*) collision, as a function of the impact energy. Solid red line: present CTMC results, solid blue line: present QCTMC results, green circles: QMOCC results of Harel et al.^4^, black triangles: BCCIS results of Das et al.^14^, blue inverse triangles: OEDM results of Errea et al.^13^.

Figure 6 shows the present CTMC and QCTMC results of the electron capture cross sections into 5*s*, 5*p*, 5*d*, and 5*f* states of the projectile in Be^4+^ + H(1*s*) as a function of the impact energy. The obtained results are compared with QMOCC^4^, BCCIS^14^, and OEDM^13^ methods, as well. According to Fig. 6, the QCTMC method outstandingly enhances the cross sections compare to the CTMC results at impact energies lower than about 60 keV/amu. Good agreements are obtained between the present QCTMC results with the OEDM results of Errea et al.^13^ and the QMOCC results of Harel et al.^4^ in 5*s*, 5*d*, and 5*f* states of the projectile. The present CTMC and QCTMC results in all 5*l*-states agree well with the BCCIS results of Das et al.^14^ at intermediate energies. The QCTMC and CTMC cross sections are approximately matched at the impact energies greater than 100 keV/amu.

According to Figs. 3, 4, 5, and 6, the present CTMC and QCTMC results of the electron capture cross sections into specific states of the projectile in Be^4+^ + H(1*s*) are given for several typical impact energies in Table 1. As we already mentioned, the QCTMC cross sections are larger compared to CTMC ones at lower incident energies. However, as the energy increases, this difference gradually decreases so that at very high energies, this difference is negligible. To explain this behavior physically, we focus on the force between the electron and the hydrogen nucleus. Typically, the net Coulomb force is applied between two bodies, which is inversely related to the square of the distance between them. Heisenberg correction term [see Eq. (6)] generates a repulsive force in the opposite direction to the Coulomb force. In this case, the attraction force between the electron and the target's nucleus decreases, increasing the electron's reactivity with the projectile's ion in the electron capture channel. Also, the long- distance of the projectile to the electron practically reduces this repulsive force's effect on the calculations (see Fig. 1).

**Table 1:** The present CTMC and QCTMC results of the electron capture cross sections into specific states of the projectile in Be^4+^ + H(1*s*).

Energy (keV/amu)	Model	Cross section (in 10^–16^ cm^2^)					
		3*s*	3*d*	4*s*	4*d*	5*s*	5*d*
1	CTMC	2.608	7.175	0.022	0.116	0.001	0.001
	QCTMC	5.698	17.81	0.686	3.370	0.017	0.056
5	CTMC	1.326	11.09	0.135	0.314	0.012	0.019
	QCTMC	3.343	15.74	0.385	3.844	0.061	0.170
10	CTMC	0.681	11.61	0.183	0.571	0.033	0.081
	QCTMC	1.757	15.46	0.276	3.364	0.084	0.316
35	CTMC	0.209	6.780	0.112	1.652	0.062	0.464
	QCTMC	0.288	6.469	0.158	2.275	0.087	1.024
55	CTMC	0.117	3.109	0.077	1.220	0.054	0.623
	QCTMC	0.144	2.787	0.089	1.161	0.055	0.658
70	CTMC	0.081	1.793	0.052	0.797	0.036	0.450
	QCTMC	0.089	1.576	0.062	0.705	0.039	0.401
90	CTMC	0.052	0.879	0.033	0.401	0.022	0.263
	QCTMC	0.040	0.569	0.028	0.405	0.023	0.212
200	CTMC	0.007	0.039	0.005	0.024	0.003	0.015
	QCTMC	0.004	0.039	0.003	0.028	0.002	0.0187

On the other hand, the passing projectile ion at low energies causes the extension of the interaction time. Therefore, the effect of these factors increases the cross section at low energies in the QCTMC model. Also, the interaction time is shorter at high energies. Furthermore, due to the small Heisenberg repulsive force, the correction term gradually loses its effects; therefore, the CTMC and QCTMC results are approximately the same.

## Discussions

The electron capture cross sections into *n* = 3, 4, 5, 6, 8, 10 and *nl* = 3*l*, 4*l*, 5*l* states of the projectile have been presented in Be^4+^  + H(1*s*) in the framework of CTMC and QCTMC methods. For the determination of the cross sections 10^7^ trajectories were calculated for each impact energies. We found that the QCTMC cross sections are higher than the CTMC ones at low energies. We have used the previous AOCC, QMOCC, BCCIS, and OEDM quantum–mechanical approaches for comparison with our present data. Including the potential correction term to mimic the Heisenberg uncertainty principle in the classical Hamiltonian, we have shown that our QCTMC capture cross sections into the projectile states, *n* = 3, 5 and *nl* = 3*s*, 3*p*, 3*d*, 4*s*, 4*p*, 5*s*, 5*d*, 5*f* are in excellent agreement with quantum–mechanical results. We believe that our model, with its simplicity, can be an alternative way to calculate accurate cross sections and maybe can replace the results of the quantum–mechanical models, where the quantum mechanical calculations become complicated.

## Methods

The QCTMC model, in principle, takes into account the Heisenberg and Pauli constraints in adding a correction term into the standard original Hamiltonian^16^. For hydrogen atom, which has only one electron, the Pauli correction can automatically neglect. Therefore, the quasi classical Hamiltonian consists of correction potential, *V*_*H*_, inspired by the Heisenberg principles can be written as:

2 $${H}_{QCTMC}={H}_{0}+{V}_{H}$$

where,

3 $${V}_{H}=\sum_{n=a.b}\sum_{i=1}^{N}f\left({r}_{ni} .{p}_{ni};{\xi }_{H} . {\alpha }_{H}\right)$$

*a* and *b* denote the nuclei, and the *i* index the electrons. *r* and *p* are the distance and momentum of an electron with respect to a nucleus, which is defined as follows:

4 $${r}_{ab}={r}_{b}-{r}_{a}$$

5 $${p}_{ab}=\frac{{m}_{a}{p}_{b}-{m}_{b}{m}_{a}}{{m}_{a}+{m}_{b}}$$

The Heisenberg correction function is expressed as^16^

6 $$f\left({r}_{ab} .{p}_{ab};{\xi }_{H} . {\alpha }_{H}\right)=\frac{{\xi }_{H}}{4{\alpha }_{H}{r}_{ab}^{2}{\mu }_{ab}}exp\left\{{\alpha }_{H}\left[1-{\left(\frac{{r}_{ab}{p}_{ab}}{{\xi }_{H}}\right)}^{4}\right]\right\}$$

where subscripts *a* and *b* indicate pairs of particles with reduced mass ${\mu }_{ab}$. The parameter ${\xi }_{H}$ reflects the size of the core while ${\alpha }_{H}$ is a hardness parameter. These parameters universally are used where ${\alpha }_{H}$ = 4 and ${\xi }_{H}$ = 0.9428, respectively^19–21^. The Heisenberg potential between the target electron and both target core and projectile (p; projectile, e; electron, T; target) are defined as follows:

7 $$f\left({\overrightarrow{r}}_{pe} .{\overrightarrow{P}}_{pe};{\varepsilon }_{H} .{\alpha }_{H}\right)=\frac{{{\xi }_{H}}^{2}}{4{\alpha }_{H}{\overrightarrow{r}}_{pe}^{2}{\mu }_{pe}}exp\left\{{\alpha }_{H}\left[1-{\left(\frac{{\overrightarrow{r}}_{pe}{\overrightarrow{P}}_{pe}}{{\xi }_{H}}\right)}^{4}\right]\right\}$$

8 $$f\left({\overrightarrow{r}}_{Te} .{\overrightarrow{P}}_{Te};{\varepsilon }_{H} .{\alpha }_{H}\right)=\frac{{{\xi }_{H}}^{2}}{4{\alpha }_{H}{\overrightarrow{r}}_{Te}^{2}{\mu }_{Te}}exp\left\{{\alpha }_{H}\left[1-{\left(\frac{{\overrightarrow{r}}_{Te}{\overrightarrow{P}}_{Te}}{{\xi }_{H}}\right)}^{4}\right]\right\}$$

The total cross sections are computed with the following formula:

9 $$\sigma =\frac{2\pi {b}_{max}}{{T}_{N}}\sum_{j}{b}_{j}^{(i)}$$

and the statistical uncertainty of the cross sections is given by:

10 $$ \Delta \sigma  = \sigma \left( {\frac{{T_{N}  - T_{N}^{{\left( i \right)}} }}{{T_{N} T_{N}^{{\left( i \right)}} }}} \right)^{{1/2}}  $$

where *T*_*N*_ is the total number of trajectories calculated for impact parameters less than *b*_*max*_, $T_{N}^{(i)}$ is the number of trajectories that satisfy the criteria for the corresponding final channels (electron capture), and *b*_*j*_^*(i)*^ is the actual impact parameter for the trajectory corresponding electron capture processes.

In the classical approaches, the classical principal (*n*_*c*_) and the orbital angular momentum (*l*_*c*_) quantum numbers are defined by

11 $$ n_{c} = Z_{T} Z_{e} \left( {\frac{{\mu_{Te} }}{2U}} \right)^{1/2} $$

12 $${l}_{c}=\sqrt{{m}_{e}\left[{\left(x\dot{y}-y\dot{x}\right)}^{2}+{\left(x\dot{z}-z\dot{x}\right)}^{2}+{\left(y\dot{z}-z\dot{y}\right)}^{2}\right]}$$

where ${\mu }_{Te}$ is the reduced mass of the target nucleus and the target electron. *x*, *y*, and *z* are the Cartesian coordinates of the electron relative to the nucleus and $\dot{x}$, $\dot{y}$, and $\dot{z}$ are the corresponding velocities. The classical values of *n*_*c*_ are “quantized” to a specific level *n*^22^ if they satisfy the relation:

13 $${\left[\left(n-1\right)\left(n-1/2\right)n\right]}^{1/3}\le {n}_{c}\le {\left[\left(n+1\right)\left(n+1/2\right)n\right]}^{1/3}$$

Since *l*_*c*_ is uniformly distributed for a given *n* level^23^, the quantal statistical weights are reproduced by choosing bin sizes such that

14 $$l\le \frac{n}{{n}_{c}}{l}_{c}\le l+1$$

where *l* is the quantum–mechanical orbital angular momentum.
